# Comparative support for the expensive tissue hypothesis: Big brains are correlated with smaller gut and greater parental investment in Lake Tanganyika cichlids

**DOI:** 10.1111/evo.12556

**Published:** 2014-12-17

**Authors:** Masahito Tsuboi, Arild Husby, Alexander Kotrschal, Alexander Hayward, Séverine D Buechel, Josefina Zidar, Hanne Løvlie, Niclas Kolm

**Affiliations:** 1 Evolutionary Biology Centre, Department of Ecology and Genetics/Animal Ecology, Uppsala UniversityNorbyvägen 18D, SE-75236, Uppsala, Sweden; 3 Department of Biosciences, University of HelsinkiPO Box 65, FI-00014, Helsinki, Finland; 4 Department of Zoology/Ethology, Stockholm UniversitySvante Arrhenius väg 18B, SE-10691, Stockholm, Sweden; 5 ETH Zürich Institute of Integrative Biology (IBZ)Universitätsstrasse 16, 8092, Zürich, Switzerland; 6 IFM Biology, Linköping UniversityCampus Valla, SE-58183, Linköping, Sweden

**Keywords:** Brain evolution, constraints, encephalization, phylogenetic comparative methods, the expensive tissue hypothesis, trade-offs

## Abstract

The brain is one of the most energetically expensive organs in the vertebrate body. Consequently, the energetic requirements of encephalization are suggested to impose considerable constraints on brain size evolution. Three main hypotheses concerning how energetic constraints might affect brain evolution predict covariation between brain investment and (1) investment into other costly tissues, (2) overall metabolic rate, and (3) reproductive investment. To date, these hypotheses have mainly been tested in homeothermic animals and the existing data are inconclusive. However, there are good reasons to believe that energetic limitations might play a role in large-scale patterns of brain size evolution also in ectothermic vertebrates. Here, we test these hypotheses in a group of ectothermic vertebrates, the Lake Tanganyika cichlid fishes. After controlling for the effect of shared ancestry and confounding ecological variables, we find a negative association between brain size and gut size. Furthermore, we find that the evolution of a larger brain is accompanied by increased reproductive investment into egg size and parental care. Our results indicate that the energetic costs of encephalization may be an important general factor involved in the evolution of brain size also in ectothermic vertebrates.

The brain is one of the most energetically expensive organs in the vertebrate body (Mink et al. 1981). The large amount of energy required to maintain brain tissue should therefore impose serious constraints on brain size evolution (Striedter 2005), despite the cognitive benefits of having a large brain (Jerison 1973; Striedter 2005; Kotrschal et al. 2013a,b). Three major hypotheses have been proposed to explain the cost of encephalization (i.e., an evolutionary increase in relative brain size, Jerison 1973). First, the direct metabolic constraints hypothesis suggests that due to the energetic cost of maintaining brain tissue, overall metabolic rate at resting (i.e., basal metabolic rate, BMR) should be positively associated with brain size (Martin 1981; Isler and van Schaik 2006b; Isler and van Schaik 2009). Second, the expensive tissue hypothesis (ETH) argues that the cost of encephalization should be compensated by a reduction in the size of other expensive organs (Aiello and Wheeler 1995; Isler and van Schaik 2009). Third, a recently proposed extension of the original ETH is the “energy trade-off hypothesis” (Isler and van Schaik 2006a) that suggests that the cost of increased brain size can be met through a series of trade-offs with costly aspects other than tissue investment such as body maintenance (Isler and van Schaik 2006b), locomotion (Navarrete et al. 2011), and reproduction (Isler and van Schaik 2009). So far, these three major hypotheses have been investigated only in a handful of taxa and mainly in homeothermic animals (i.e., mammals and birds). Interestingly, a recent experimental study using guppies (*Poecilia reticulata*) that had been artificially selected for divergence in brain size found that increased brain size led to accompanying reduction in fecundity and gut size (Kotrschal et al. 2013a). This suggests that the evolutionary implications of energetic constraints could be similar for both homeothermic and ectothermic vertebrates.

Currently, comparative studies that have tested energetic constraints on brain size evolution have provided inconclusive evidence. Since originally being proposed by Aiello and Wheeler (1995), the ETH has formed a controversial hypothesis partly because the results have been dependent on analytical details (Aiello et al. 2001), and partly because results vary between taxa. For instance, Isler and van Schaik (2006a) reanalyzed the original dataset from Aiello and Wheeler (1995) with phylogenetic correction and confirmed the ETH in primates. However, the negative correlation between brain size and gut size was later refuted with a more comprehensive dataset across mammals (Navarrete et al. 2011). Moreover, investigations using birds (Isler and van Schaik 2006a) and bats (Jones and MacLarnon 2004) have failed to find any correlation between brain size and gut size. Hence, except for one small-scale comparative study (Kaufman et al. 2003) the “brain versus gut” trade-off is so far not supported by data at the across-species level. Nevertheless, the ETH has gained some support in other forms, including a trade-off against gonads (Pitnick et al. 2006, but see Lemaitre et al. 2009), fat storage (Navarrete et al. 2011), and muscle (Isler and van Schaik 2006a; Muchlinski et al. 2012). Isler and van Schaik (2009) recently proposed that these apparent discrepancies between studies could be reconciled under a broader framework that argues that the increased cost of a large brain can be met by changing the allocation of any energetically costly aspects (the expensive brain framework). However, the expensive brain framework has so far been applied almost exclusively to homeothermic vertebrates. Overall, to assess the generality of the described hypotheses concerning vertebrate brain evolution, an independent examination with a novel dataset is warranted, ideally focusing on ectothermic vertebrates.

There are several reasons why the cost of maintaining brain tissue should impact on brain size evolution in ectothermic vertebrates. For example, the brain tissue of ectothermic animals is actually more costly in relative terms than in homeothermic animals, because ectotherms must maintain brain tissue that is as costly as any homeotherm's brain tissue (Mink et al. 1981; Nilsson 1996) despite having a more than 10-fold lower whole-body metabolic rate (Nilsson 1996). Also, in ectothermic animals, the metabolic activity of brain tissue is less responsive to ambient temperature than in the case for the whole body metabolism (Peterson and Anderson 1969; Clarke 1983; Heath 1988). This imposes an energy constraint on maintaining and developing larger brains in cold environments. Additionally, the example of the extraordinarily large brain in the elephant nose fish, *Gnathonemus petersii*, is indeed associated with a reduction in intestine and stomach size (Kaufman et al. 2003). This implies that energy constraints on brain size evolution exist at least in this highly encephalized tropical species. As a last piece of support, recently developed guppy brain size selection lines revealed a cost of increased brain size through reduction in gut size and offspring number (Kotrschal et al. 2013a). Together these examples suggest that implications of energetic constraints and tissue size are not limited to mammalian and avian taxa, but should also play a key-role in determining patterns of brain size diversification across ectothermic vertebrates.

The cichlids of the African Great Lakes have undergone rapid diversification and form one of the classic examples of adaptive radiation (Schluter 2000). The remarkable ecological diversity of this lineage offers an appealing opportunity to test the three key hypotheses concerning energetic constraints on brain size evolution described above. In aquatic ectothermic organisms, BMR decreases with increasing water depth (Smith and Hessler 1974; Childress 1975; Torres et al. 1979; Seibel and Drazen 2007). According to the direct metabolic constraints hypothesis, we thus predict that the water depth a species inhabits is negatively associated with its brain size. Also, the length of the digestive tract in Lake Tanganyika cichlids is extremely variable in relation to diet (Fryer and Iles 1972; Wagner et al. 2009; Kotrschal et al. 2014). Algae eating species have considerably longer guts compared to species that primarily consume protein rich food material, reflecting the greater digestive processing time of vegetable matter compared to meat (Horn 1989). Based on the ETH, we therefore predict that a smaller brain should accompany a long gut and vice versa. Moreover, the level of reproductive investment, manifested mainly by egg size, clutch size, and care duration in Lake Tanganyika cichlids, shows considerable variation across species (Keenleyside 1991; Goodwin et al. 1998; Kolm et al. 2006). According to the patterns reported in previous comparative tests of the energy trade-off hypothesis, we predict that large-brained cichlid species produce larger eggs to fuel the developmental cost of large-brained juveniles, smaller clutches to compensate the energetic requirement for large eggs, and have longer periods of parental care.

The goal of this study was thus to test a series of key predictions arising from the expensive brain framework (Table 1). By exploiting the dramatic diversity in ecology, life history, and brain size among Lake Tanganyika cichlids, we attempt to assess if the energetic costs of encephalization play an important role in brain size diversification within ectothermous animals with implications across vertebrates in general.

**Table 1:** List of hypotheses concerning the energetic cost of brain evolution and predictions following from each hypothesis.

Hypothesis	Variables tested	Predicted link to brain size	Motivations for predictions
(1) Direct metabolic constraints	Depth as a proxy of BMR	Negative	Larger brains require higher BMR (Martin 1981; Isler and van Schaik 2006b)
(2) Expensive tissue	Gut length	Negative	The trade-off between investment into different energetically expensive organs (Aiello and Wheeler 1995; Kaufman et al. 2003)
(3) Energy trade-off	Egg size	Positive	Species with larger brains invest more into each egg to fulfill increased energetic requirements for offspring with larger brains (Isler and van Schaik 2009; Barton and Capellini 2011)
	Clutch size	Negative	Species with larger brains compensate for increased investment into a larger brain by reducing egg number per brood (Isler and van Schaik 2009; Weisbecker and Goswami 2010)
	Care duration	Positive	Species with larger brains prolong care duration because of longer developmental time in offspring with larger brains (Foley and Lee 1991)

## Materials and Methods

### Data Collection

Sampling was conducted in the southern part of Lake Tanganyika near Mpulungu, Zambia, during September 2012 in compliance with Zambian legislation. Specimens were sacrificed using an overdose of benzocaine. Body mass was recorded to the nearest 1.0 g and the head was severed and preserved in 4% paraformaldehyde-phosphate buffer solution. The fresh digestive tract was removed from the body cavity of each specimen, the intestines were carefully uncoiled in a water bath, and intestine length was measured as the distance from the posterior end of the stomach to the anus to the nearest millimeter using a standard ruler. Sex was identified by careful examination of the gonads of each individual and only sexually mature adults were used in the analyses. Whole brains were obtained from dissected heads following fixation and weighed using a Precisa 125A electronic scale (precision = 0.01 mg; Precisa Instruments AG, Switzerland). All cranial nerves, optic nerves, and meningeal membranes were removed and the brain was severed from the spinal cord immediately posterior of the dorsal medulla. To avoid biases in brain morphology due to different fixation times, all brain measurements were made within a five-day period following 10 months of tissue preservation (storage at 4°C). We followed the brain dissection and measurement protocol performed by Gonzalez-Voyer et al. (2009), enabling us to pool our data on brain size with previously published data (Gonzalez-Voyer et al. 2009). The pooling of two datasets should not bias our study because an analysis of covariance (ANCOVA) with log_10_ transformed brain size as a response variable and the origin of data (i.e., either from Gonzalez-Voyer et al. 2009 or from our own sampling) and log_10_ transformed body weight as explanatory variables showed no effect (*Ssq* = 0.20, *F*_353,1_ = 1.12, *P* = 0.29). The pooled dataset on brain size consisted of 707 individuals across 71 species, among which 490 individuals across 54 species were from our own sampling, representing all but one tribe of Lake Tanganyika cichlid, the *Boulengerochromini* (Salzburger et al. 2002). Our data covered 14 species across all four tribes that occur deeper than 100 m, which is close to the anoxic layer of the lake (about 130 m depth at the bay near Mpulungu, Takahashi et al. 2007), and includes “the rarest Tanganyikan cichlid”, *Baileychromis centropomoides* (Konings 2005).

### Ecological Data

We collected ecological and life-history data from books and published literature for the 71 species included (Supporting Information Table S1). Description of the range of water depths that each species occupies was obtained from the literature (Supporting Information Table S1) and the average of the shallowest and the deepest recorded depth was calculated as a proxy of inhabiting water depth. We set the deepest possible inhabiting depth for our samples to 130 m, at which level the anoxic layer starts at our sampling location (Takahashi et al. 2007). Because the difference in nutritional composition of the diet might influence total energy budget and confound the relationship between brain and gut size (Fish and Lockwood 2003; Wagner et al. 2009; Allen and Kay 2012), we also included information on trophic guild in our analyses. Based on the gut contents and field observations documented in books and published literature (Supporting Information Table S1), we assigned our species to five major trophic guilds found in Lake Tanganyika cichlids: algivores, invertivores, scale-eaters, zooplanktivores, and piscivores. Egg size was obtained as egg diameter (mm), and we approximated egg volume as a sphere (*V* = 4π*r*^3^/3). Finally, information on clutch size (the number of eggs per brood) and care duration (the number of days after the eggs were fertilized until the juveniles become independent) was also collected from the literature. Ecological and life-history variables for each species and other sources of information are summarized in Supporting Information Table S1.

### Statistical Analyses

**Figure 1:**
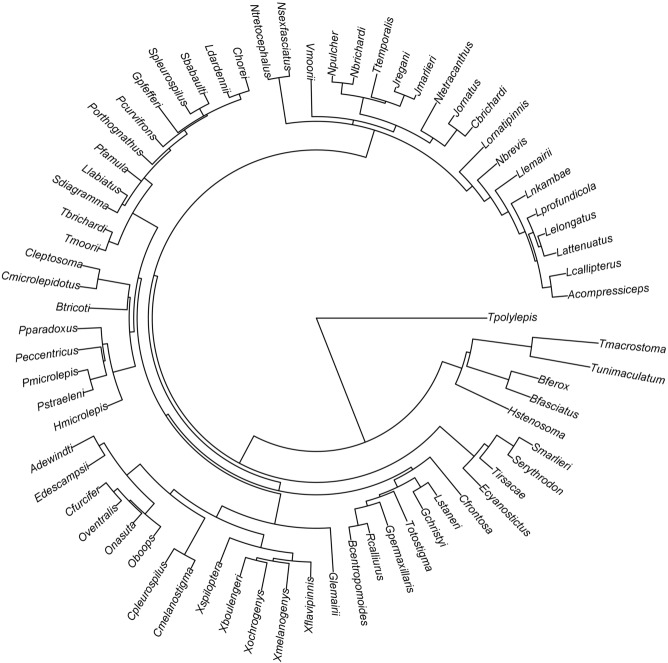
A molecular phylogeny of 71 Lake Tanganyika cichlids used in the present study. Species names are given with a capitalized first letter for the genus followed by the species name. For details regarding phylogenetic reconstruction, please see Amcoff et al. 2013.

All statistical analyses were performed in the R statistical environment (R Development Core Team 2011). Our phylogenetic tree is based on mitochondrial sequences downloaded from Genbank (Fig. 1, for details on phylogeny reconstruction, see Amcoff et al. 2013). We assessed the phylogenetic signal (i.e., the λ statistic) in our data through a maximum likelihood estimation using the phytools package (Revell 2012), and identified strong phylogenetic signals for all but one trait examined in our study (λ: body mass = 0.99, brain mass = 0.96, gut length = 0.96, depth = 0.99, trophic guild = 0.99, egg size = 0.91, clutch size = 0.75, care duration = 0.003). Therefore, we controlled for phylogeny in our analyses, but also ran additional nonphylogenetic analyses for models that included care duration. To control for shared ancestry and within-species variation simultaneously, we ran a Bayesian generalized mixed effect model (GLMM) in a phylogenetic framework using a Bayesian approach based on a Markov Chain Monte Carlo (MCMC) algorithm (Hadfield and Nakagawa 2010) implemented in the package MCMCglmm (Hadfield 2010). All continuous variables were log_10_ transformed before analyses. In all analyses, the response variable was brain size, and a main factor (i.e., water depth, gut length, egg size, clutch size, or care duration) was included as an independent variable. Body mass and sex were included as covariates in all models to control for their effect on brain size. The proposed trade-off between the size of different organs assumes an equal overall energy budget across species (van Noordwijk and de Jong 1986; Aiello and Wheeler 1995). Thus, in our assessment of the relationship between brain size and gut size, we included living water depth because it is strongly correlated with BMR in fishes (Smith and Hessler 1974; Childress 1975; Torres et al. 1979; Seibel and Drazen 2007). As mentioned above, trophic guild is a strong predictor of gut length (Wagner et al. 2009), and thus included in this model as a covariate. Overall, we constructed five separate multiple regression models, each testing one of our five predictions arising from the three current hypotheses regarding brain size evolution (Table 1). For each model, we calculated the phylogenetic signal *H*^2^ that is equivalent to λ of a phylogenetic generalized least square (PGLS) model (Housworth et al. 2004; Hadfield and Nakagawa 2010). Sample size differed slightly between models due to the heterogeneity in availability of ecological information. In all analyses, models were run using a flat improper prior on the residual variance and a parameter-expanded prior on the phylogeny. Parameter expansion resulted in a scaled *F* distribution with numerator and denominator degrees of freedom set to 1 and a scale parameter of 1000. Employment of parameter-expanded priors improved chain mixing. Models were run for 800,000 iterations with a burn-in of 100,000 and a thinning interval of 250. All chains mixed well and had low autocorrelation between successive samples of the posterior distribution (<0.1). We also formally tested chain mixing using the Heidelberg criteria (Heidelberger and Welch 1983), for which all models passed.

## Results

Table 2 summarizes the result of the three main predictions in our study. Note that brain size as discussed in the following section refers to relative brain size. This is because our interpretations are all based on the partial correlation coefficient between brain size and the variable of interest within a multiple regression model, including body mass as a covariate. We found strong phylogenetic signal in all our models (*H*^2^; 0.879–0.979, Table 2), indicating that our models require phylogenetic corrections. To evaluate the direct metabolic constraints hypothesis, we tested if water depth is associated with brain size. We found that the water depth at which a species resides was not significantly associated with brain size (*n*_individual_ = 707, *n*_species_ = 71, post. mean = 0.005, 95% credible interval = −0.014 to 0.026, *P* = 0.59). Therefore, the direct metabolic constraints hypothesis was not supported by our data.

**Table 2:** Bayesian statistics for multivariate models with phylogeny as a random factor to test the ETH in Lake Tanganyika cichlids.

Hypothesis tested	Parameter		Sample size Individual/species	*H*^2^	Posterior mean	*P*
(1) Direct metabolic constraints	Depth		707/71	0.960^(0.950, 0.977)^	0.005^(−0.014, 0.026)^	0.59
(2) Expensive tissue	Gut length		490/54	0.971^(0.961, 0.984)^	−0.013^(−0.022, −0.003)^	**0.009**
	Depth				−0.006^(−0.038, 0.026)^	0.70
	Trophic guild	Inve.			0.046^(−0.030, 0.119)^	0.23
		Pisc.			0.064^(−0.048, 0.180)^	0.29
		Scal.			−0.033^(−0.207, 0.170)^	0.72
		Zoop.			−0.019^(−0.151, 0.108)^	0.75
(3) Energy trade-off	Egg size		461/43	0.964^(0.948, 0.975)^	0.030^(0.001, 0.057)^	**0.04**
	Clutch size		707/71	0.979^(0.971, 0.987)^	0.015^(−0.006, 0.036)^	0.16
	Care duration		373/37	0.879^(0.818, 0.937)^	0.026^(0.006, 0.043)^	**0.007**

The response variable is log_10_-transformed brain mass in both models. Sample size, *H*^2^, posterior mean, and *P*-value for each parameter are presented. The 95% lower credibility interval followed by the upper credible interval is also given as a superscript for *H*^2^ and posterior mean. Note that the posterior mean for continuous variables (i.e., body mass, depth, and gut) represents the partial regression coefficient, whereas the posterior mean for trophic guild represents the differential intercept coefficient comparing each category with algivore.

Abbreviations for each level of trophic guild are as follows: Inve. = invertivore; Pisc. = piscivore; Scal. = scale eater; Zoop = zooplanktivore.

All analyses also included sex and body mass as covariates, for which the results are provided in Supporting Information Table S2. Statistically significant values are indicated in bold font.

**Figure 2:**
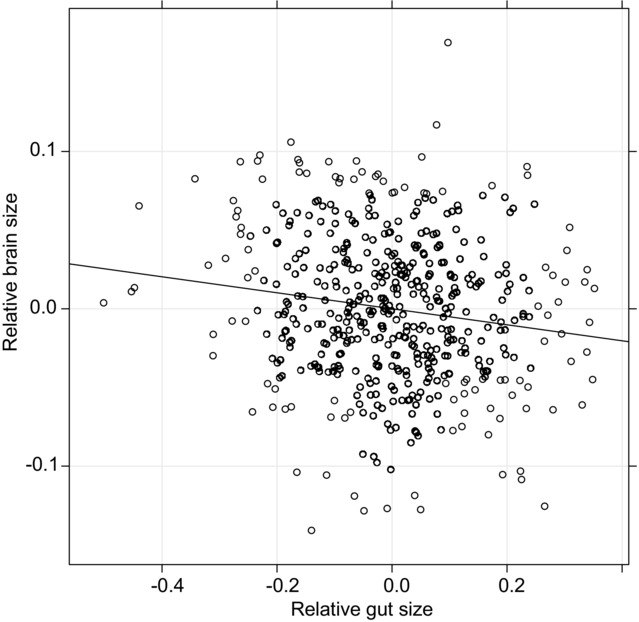
The relationship between brain and gut size. Values on the x-axis are residuals from the linear regression of log_10_ gut length as a dependent variable and log_10_ body mass, log_10_ depth, and sex as independent variables (relative gut size). Values on the y-axis are residuals from the linear regression of log_10_ brain mass as a dependent variable and log_10_ body mass, log_10_ depth, and sex as independent variables (relative brain size). The tribe to which each species are assigned was included as a random effect for calculating values in both axes. The least squares regression lines are also provided. Note that each datapoint represents each individual specimen included in the study (see Materials and Methods for details). The values are presented without phylogenetic correction for visualization purposes only, while all statistical analyses controlled for the effect of phylogeny (for details of the statistics, see Table 2).

Next, we examined the ETH. We found that brain size and gut size were negatively correlated (*n*_individual_ = 490, *n*_species_ = 54, post. mean = −0.013, 95% credible interval = −0.022 to −0.003, *P* = 0.009, Fig. 2). Inclusion of the interaction term between gut length and trophic guild in the model led to an increase in the deviance information criterion (ΔDIC = 6.27). This indicates that the model provided a better fit to our data without inclusion of the interaction term. Thus, our analyses provide support for a fundamental prediction of the ETH; a negative association between brain and gut size (Aiello and Wheeler 1995).

**Figure 3:**
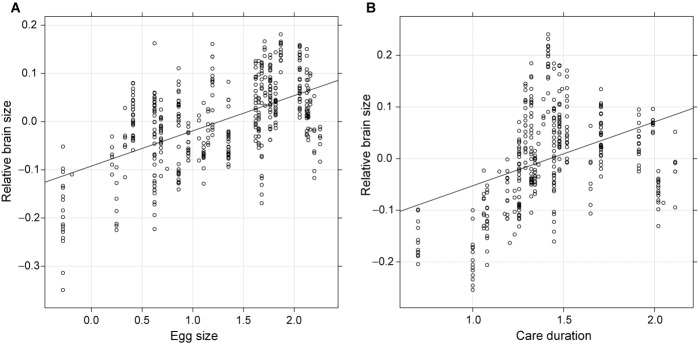
The relationship between brain and egg size (A), and brain size and care duration (B). Values on the y-axis are residuals from the linear regression of log_10_-transformed brain mass as the dependent variable, and log_10_ body mass as the independent variable (relative brain size). Note that each datapoint represents each individual specimen included in the study (see Materials and Methods for details). The values are presented without phylogenetic correction for visualization purposes only, while all analyses performed controlled for phylogeny (for details of the statistics, see Table 2).

Finally, we assessed the energy trade-off hypothesis. We found that egg size (*n*_individual_ = 461, *n*_species_ = 43, post. mean = 0.030, 95% credible interval = 0.001–0.057, *P* = 0.04, Fig. 3A) and care duration (*n*_individual_ = 373, *n*_species_ = 37, post. mean = 0.026, 95% credible interval = 0.006–0.043, *P* = 0.007, Fig. 3B) were both positively associated with brain size, supporting two predictions arising from the energy trade-off hypothesis. The associations between egg size, clutch size, and care duration in Lake Tanganyika cichlids (Kolm et al. 2006) do not confound our results because a multiple regression analysis, including egg size, clutch size, and care duration in the same model yielded equivalent results (*n*_individual_ = 269, *n*_species_ = 24, egg size; post. mean = 0.036, 95% credible interval = 0.004–0.062, *P* = 0.02, clutch size; post. mean = 0.015, 95% credible interval = −0.019 to 0.049, *P* = 0.36, care duration; post. mean = 0.020, 95% credible interval = 0.001–0.039, *P* = 0.046). Visual examination suggested that the relationship between brain size and care duration had a nonlinear relationship (Fig. 3B). However, nonlinearity was not statistically supported because inclusion of a quadratic term into the model did not improve model fit (ΔDIC = −0.196) and the quadratic term was not statistically significant (*n*_individual_ = 373, *n*_species_ = 37, post. mean = – 0.083, 95% credible interval = – 0.279 to 0.115, *P* = 0.39). Nonphylogenetic analyses with care duration did not influence our conclusions (Supporting Information Table S3).

## Discussion

Here, we investigated large-scale macroevolutionary patterns of multiple energetic constraints on brain size evolution for the first time outside of homothermous taxa. We found a negative association between brain size and gut size after controlling for several potential confounding ecological factors and phylogenetic nonindependence, supporting the original “brain versus gut” prediction arising from the ETH (Aiello and Wheeler 1995). Moreover, we found that the evolution of a larger brain was accompanied by an increase in egg size and prolongation of the parental care period. Thus, our study supports the existence of energetic constraints as important factors influencing across-species patterns of brain size diversification in cichlids. More widely, our findings imply that energetic constraints of encephalization are not limited to homeothermic vertebrates, but are also important in ectothermic vertebrate brain size evolution. Below, we discuss our results in more detail in light of the three main hypotheses addressed.

### The Direct Metabolic Constraints Hypothesis

The direct metabolic constraints hypothesis predicts that the cost of increased brain size can be met by increasing overall energy turnover (Martin 1981). Although studies in mammals have found support for this hypothesis (Martin 1998; Isler and van Schaik 2006b), studies on birds (Isler and van Schaik 2006a) and bats (Jones and MacLarnon 2004) did not find a relationship between BMR and brain size. Our study used the water depth at which a species lives as a proxy for BMR because it is strongly correlated with BMR in fishes (Smith and Hessler 1974; Childress 1975; Torres et al. 1979; Seibel and Drazen 2007). Similar to comparative studies performed on birds and bats, we failed to find support for the predicted relationship between brain size and BMR (Table 2). We propose four possible explanations for this result. First, the physiological basis of the nearly ubiquitous relationship between metabolic rate and water depth in aquatic organisms is not yet completely understood (Childress 1995). Water temperature (Clarke 1983), oxygen level (Childress and Seibel 1998), and water pressure (Somero and Siebenaller 1979) may all influence metabolic activity and so could have confounded our result. Thus, employing direct measurements of BMR in future studies may provide more accurate data to test the direct metabolic constraints hypothesis. Second, unlike mammals and birds, teleost fishes undergo life-long neurogenesis (Zupanc 2006). Even though we selected only sexually mature adults for our analyses to minimize potential noise from brain size allometry, variation in age within adult individuals might have introduced variation to our data. Third, the relationship between BMR and brain size was originally proposed after the observation of an isometric relationship between neonatal brain size and maternal metabolic rate (Martin 1981). If maternal metabolic turnover puts an upper boundary on fetal brain size (Martin 1996), the pattern should be observed most commonly in animals with prolonged gestation through a placenta because it results in a more direct physiological contact between mother and offspring. Indeed, the relationship between BMR and brain size has so far been supported in several placental mammalian clades (Martin 1998; Isler and van Schaik 2006b; Weisbecker and Goswami 2010), but refuted in marsupials (Weisbecker and Goswami 2010) and birds (Isler and van Schaik 2006a). Thus, the evolutionary link between brain size and BMR might be a specific pattern within placental vertebrates (but see Jones and MacLarnon 2004; Isler 2011). Finally, in various groups of fishes, the metabolic rate of skeletal muscle decreases with increasing water depth (Childress and Somero 1979; Torres and Somero 1988), while the brain maintains a constant metabolic rate across species from different depth (Childress and Somero 1979; Sullivan and Somero 1980). Therefore, our findings may indicate that metabolically less active skeletal muscles compensate for the high metabolic cost of brain tissue in deep-living cichlids. Incorporation of these four possibilities in future studies may further improve our understanding of the metabolic constraints acting on encephalization in teleost fishes.

### The Original ETH

The original prediction of the ETH is that the cost of an increase in brain size should be compensated for by a reduction in the size of other energetically expensive organs, more specifically of the gut (Aiello and Wheeler 1995; Isler and van Schaik 2009). Our investigation of the ecologically diverse Lake Tanganyika cichlids shows that larger brains are accompanied by shorter guts that supports the ETH in its original form (Aiello and Wheeler 1995). Because a recent reexamination of the data from Aiello and Wheeler (1995) failed to support the original idea (Navarrete et al. 2011), our dataset is currently the only data that directly supports the brain–gut trade-off in animals at the across-species level. Previously, only a small-scale nonphylogenetic comparative study on the elephant nose fish *G. petersii* (Kaufman et al. 2003) and a recent experimental study on the guppy (Kotrschal et al. 2013a) reported a trade-off between brain size and gut size. Our result thus adds support at the across-species level that the energy requirements of encephalization can be compensated for by reducing gut size in ectothermic animals. The lack of prior studies on ectothermic animals could partly have arisen because the ETH was originally proposed as a specific explanation for the extraordinarily large brains of several anthropoid primates and humans (Aiello and Wheeler 1995). In contrast to these large-brained groups in which brain mass corresponds to 1–2% of total body mass (Striedter 2005), Lake Tanganyika cichlids have relatively small brains that, on average, correspond to only 0.07% of body mass in our dataset. However, based on our results, we propose that energetic constraints under trade-offs in investment into organs of large energetic cost play an important role irrespective of relative brain size, at least up to the level identified in Lake Tanganyika cichlids. Thus, such constraints may be much more generally applicable across vertebrates than is currently appreciated.

### The Energy Trade-Off Hypothesis

A growing number of studies suggest that the energetic requirements of encephalization can be met by changing energy allocation in reproductive investment (Isler and van Schaik 2009; Isler and Van Schaik 2014). We find that egg size and care duration are both positively associated with brain size in Lake Tanganyika cichlids. These results are in line with previous studies in mammals showing that species with larger brains produce larger neonates (Isler and van Schaik 2009) and have prolonged gestation and lactation periods (Pagel and Harvey 1988, 1990; Jones and MacLarnon 2004; Isler and van Schaik 2009; Weisbecker and Goswami 2010; Barton and Capellini 2011). Our analyses thus indicate that the energetic requirements for evolving large brains can be met through two complementary strategies: producing larger eggs and/or prolonging the duration of parental care in cichlids. Large eggs permit both larger embryos and higher energy reserves (Hutchings 1991) leading to a number of advantages for juvenile fish, such as increased growth rate, higher resistance against starvation, and overall improved survivability (Miller et al. 1988; Einum and Fleming 1999; Segers and Taborsky 2011). Therefore, large eggs in large-brained cichlids could be an evolutionary consequence of fulfilling the increased energetic costs necessary to develop large brains. The second potential strategy to increase brain size is to prolong the duration of parental care. Unlike gestation and lactation in mammals, cichlid fishes commonly do not provide nutrition directly to their offspring during brood care (Sefc 2011). Instead, parental care essentially serves as a protection against predators (Nagoshi 1987; Taborsky and Foerster 2004; Sefc 2011), which enables juvenile fish to spend more time feeding outside their shelter (Lima and Dill 1990; Foam et al. 2005). A few species of cichlid, however, are known to feed their offspring directly (Yanagisawa and Ochi 1991; Schurch and Taborsky 2005; Ota and Kohda 2014). Therefore, prolonged parental care can both directly and indirectly enhance the nutritional condition of juveniles to fuel the energetic cost of developing large brains. We did not find any reduction in clutch size in species with larger brains, but such a pattern has been documented within a single species, the guppy (Kotrschal et al. 2013a). Even though within-species patterns are not necessarily comparable to between-species patterns (Gonda et al. 2013), we speculate that the discrepancy between this previous result and our current findings might be due to reproductive differences between Lake Tanganyika cichlids and the guppy. Although Tanganyikan cichlids lay eggs and guard their fry extensively (Sefc 2011), guppies give birth to live, immediately independent young (Evans et al. 2011). Indeed, Mull et al. (2011) demonstrated that live bearing species have larger brains than egg-laying species within the chondrichthyans, indicating a strong impact of reproductive strategy on brain size evolution. Future studies comparing brain size across a variety of reproductive strategies could thus provide further insights into the factors that underlie potential trade-offs that affect brain size in fishes.

To conclude, our study demonstrates macroevolutionary patterns in support of the existence of energetic constraints on brain size evolution outside of homeothermic vertebrates, especially regarding the ETH. The hypothesis was first formulated to explain hominid evolution (Aiello and Wheeler 1995) and subsequent attention has primarily focused on large-brained, homeothermic animals. However, a growing number of studies across a variety of taxa suggest that energetic constraints acting on the evolution and maintenance of brain tissue are substantial across vertebrates (Kaufman et al. 2003; Isler and van Schaik 2006a; Weisbecker and Goswami 2010; Isler 2011; Kotrschal et al. 2013a). Our study adds significant generality to the idea that the expensive brain framework (Isler and van Schaik 2009) is a useful tool to understand macroevolutionary patterns of diversification in brain size across vertebrates.

## Data Archiving

The doi for our data is 10.5061/dryad.26pt6.

## Supporting Information

Table S1 Variables for 71 Lake Tanganyika cichlid species used in our study. **Table S2**. A complete Bayesian statistics for multivariate models with phylogeny as random factors for our test for the expensive tissue hypothesis. **Table S3**. Bayesian statistics for multivariate models without phylogenetic correction.
