# Supplementary material for: Comparative support for the expensive tissue hypothesis: Big brains are correlated with smaller gut and greater parental investment in Lake Tanganyika cichlids
Source: Evolution. 2014 Dec 17;69(1):190–200. doi: 10.1111/evo.12556 (PMC4312921; doi:10.1111/evo.12556)
Supplement: Table S1 — Variables for 71 Lake Tanganyika cichlid species used in our study. Table S2. A complete Bayesian statistics for multivariate models with phylogeny as random factors for our test for the expensive tissue hypothesis. Table S3. Bayesian statistics for multivariate models without phylogenetic correction. [file evo0069-0190-sd1.pdf]

The energetics of brain size evolution revisited in a group of ectothermic vertebrates, the Tanganyikan cichlids

Masahito Tsuboi, Arild Husby, Alexander Kotrschal, Alexander Hayward, Séverine Büchel, Josefina Zidar, Hanne Løvlie, and Niclas Kolm

Supporting Information

Additional analyses

Non-phylogenetic MCMCglmm

In our data, care duration represented a small phylogenetic signal (λ = 0.003). Because a phylogenetic correction unnecessarily decreases the type I error rate when the trait shows a weak phylogenetic signal (Freckleton et al 2002), we tested if the removal of phylogeny as a random factor from the model influences our conclusion. We used MCMCglmm package (Hadfield 2010) implemented in R (R Development Core Team 2011) to perform our additional analyses. Details of the analyses are identical to the description in the main text, but we did not apply our molecular phylogeny as a random factor.

The result of our non-phylogenetic MCMCglmm is summarized in Table S3. We found that care duration was positively associated with brain size in the single

factor model ($n_{individual}$ = 373, $n_{species}$ = 37, post. mean = 0.037, 95 % credible interval = 0.028 to 0.046, $p < 0.001$). Also, the multiple factor model where egg size, clutch size, and care duration were included in one model yielded equivalent result to a phylogenetic controlled MCMCglmm ($n_{individual}$ = 269, $n_{species}$ = 24, egg size; post. mean = 0.045, 95 % credible interval = 0.034 to 0.057, $p < 0.001$, clutch size; post. mean = 0.011, 95 % credible interval = -0.001 to 0.023, $p = 0.07$, care duration; post. mean = 0.028, 95 % credible interval = 0.020 to 0.036, $p < 0.001$). Therefore, we confirmed that our conclusions drawn from a phylogenetic Bayesian analysis is not influenced by the small phylogenetic signal in care duration.

Additional tables

To support our conclusion in the main text, we provide three additional tables and one additional figure.

References

Freckleton, R. P., P. H. Harvey, and M. Pagel. 2002. Phylogenetic Analysis and Comparative Data: A Test and Review of Evidence. The American Naturalist 160: 712-726

Hadfield, J. D. 2010. MCMC Methods for Multi-Response Generalized Linear Mixed Models: The MCMCglmm R Package. Journal of Statistical Software 33:1-22.

R Development Core Team. 2011.R: A language and Environment for Statistical Computing, version 2.13.2.R Foundation for Statistical Computing, Vienna, Austria.

Table S1: Variables for 71 Lake Tanganyika cichlid species used in our study. Sample size, body weight, gut length, brain weight, trophic guild, range of living water depth, egg diameter, clutch size, care duration, and the source of ecological information are presented. Note that body weight, gut length, and brain weight are the values only for samples that are original to our study. The '-' sign denotes that the value is missing.

Species	Sample size			Body weight	Gut length	Brain weight	Trophic guild	Depth (m -m)	Egg diameter (mm)	Clutch size	Care duration (days)	References
	Female	Male	Original to our study	(g) ± s.e.	(mm) ± s.e.	(mg) ± s.e.						
Altolamprologus compressiceps	15	5	14	88.4 ± 9.9	39.6 ± 2.7	66.0 ± 3.2	Invertivore	5-30	2.10	243	18	5, 7, 25
Aulonocranus dewindti	2	18	14	182.0 ± 4.7	105.7 ± 7.1	121.1 ± 3.1	Invertivore	1-10	4.30	27	20	7, 9
Baileychromis centropomoides	1	3	4	360.0 ± 31.5	85.0 ± 27.5	138.2 ± 7.6	Piscivore	40-100	2.80	200	-	3, 7
Bathybates fasciatus	0	11	7	464.7 ± 113	84.7 ± 11.8	157.5 ± 21.4	Piscivore	0-150	-	25	-	7
Bathybates ferox	2	4	5	599.0 ± 30.5	162.2 ± 8.3	214.8 ± 15.3	Piscivore	70-130	6.50	63	-	7
Benthochromis tricoti	2	13	13	389.6 ± 10.8	101.8 ± 7.4	162.6 ± 3.1	Zooplanktivore	20-40	2.00	12	-	7, 8
Callochromis melanostigma	1	2	0	-	-	-	Invertivore	0-5	2.00	43	-	7
Callochromis pleurospilus	0	4	0	-	-	-	Invertivore	2-2	-	33	-	7
Chalinochromis brichardi	2	10	11	108 ± 12.6	77.8 ± 3.5	51.7 ± 2.4	Invertivore	5-15	-	275	5	2, 7
Ctenochromis horei	8	8	14	142.9 ± 19.9	113.0 ± 10.0	132.3 ± 9.3	Invertivore	2-10	-	55	26	7, 17
Cyathopharynx furcifer	3	13	11	332.5 ± 40.0	213.7 ± 29.5	151.3 ± 7.4	Algivore	0-22	4.80	38	-	7, 9
Cyphotilapia frontosa	5	2	2	623.5 ± 108.5	280 ± 90	149.2 ± 10.7	Piscivore	5-70	6.70	25	45	7, 23
Cyprichromis leptosoma	2	17	13	170.6 ± 10.1	90.0 ± 4.7	98.8 ± 3.9	Zooplanktivore	3-40	4.40	6	-	7, 9
Cyprichromis microlepidotus	0	5	0				Zooplanktivore	10-40	5.10	8	-	7, 14
Ectodus descampsii	5	9	14	86.9 ± 3.7	88.4 ± 9.9	62.0 ± 1.9	Invertivore	0-25	2.90	25	21	7
Eretmodus cyanostictus	8	10	14	75.4 ± 5.9	133.9 ± 10.2	58.1 ± 2.2	Algivore	0-3	3.50	18	22	7, 10, 11
Gnathochromis permaxillaris	7	8	8	393.1 ± 53.1	208.8 ± 20.8	108.3 ± 9.3	Invertivore	30-100	2.00	65	12	3, 7, 8
Gnathochromis pfefferi	9	2	11	84.9 ± 6.1	63.1 ± 4.1	89.8 ± 4.8	Invertivore	1-15	3.10	46	21	7, 14
Grammatotria lemairii	5	3	8	809.4 ± 36.0	188.9 ± 10.4	170.5 ± 3.2	Invertivore	5-75	4.50	100	28	7, 8
Greenwoodochromis christyi	3	2	2	311.0 ± 58.0	85.5 ± 6.5	104.5 ± 6.1	Piscivore	40-130	-	150	-	7, 8
Haplotaxodon microlepis	7	12	12	480.2 ± 39.6	153.5 ± 8.6	174.8 ± 7.8	Zooplanktivore	0-20	2.40	150	51	7, 9
Hemibates stenosoma	5	1	5	492.4 ± 17.8	185.0 ± 16.8	158.5 ± 5.5	Piscivore	60-100	7.00	31	-	3, 7
Julidochromis marlieri	5	1	0	-	-	-	Invertivore	5-30	1.45	13	16	7, 19
Julidochromis ornatus	8	7	15	43.9 ± 3.3	52.7 ± 4.8	40.1 ± 1.4	Invertivore	1-40	-	25	105	1, 7, 8
Julidochromis regani	4	2	0	-	-	-	Invertivore	0-10	-	150	16	7, 8
Lamprologus callipterus	0	4	0	-	-	-	Invertivore	0-50	-	280	17	2, 7, 16, 25
Lamprologus lemairii	1	0	1	98.0	30.0	78.1	Piscivore	0-100	2.10	874	18	5, 7
Lamprologus ornatipinnis	5	8	13	21.3 ± 2.8	20.5 ± 1.5	26.0 ± 2.4	Zooplanktivore	10-100	-	15	-	7
Lepidiolamprologus attenuatus	1	0	1	101.0	36.0	60.3	Piscivore	0-20	1.07	229	112	2, 5, 7
Lepidiolamprologus elongatus	8	7	13	296.0 ± 39.2	54.2 ± 4.9	131.5 ± 8.4	Piscivore	5-50	1.70	1108	82	2, 5, 7
Lepidiolamprologus nkambae	1	5	0	-	-	-	Piscivore	3-45	-	500	-	4, 7
Lepidiolamprologus profundicola	10	5	15	503.9 ± 55.9	78.1 ± 6.6	131.6 ± 7.1	Piscivore	5-100	2.40	1275	18	5, 7
Limnochromis staneri	7	4	4	387.5 ± 106.7	210.3 ± 15.4	165.0 ± 18.8	Invertivore	15-100	2.00	200	-	3, 7
Limnotilapia dardennii	3	7	2	670.0 ± 324.0	359.5 ± 119.5	240.1 ± 42.1	Invertivore	0-50	6.00	75	-	7
Lobochilotes labiatus	6	8	13	338.4 ± 56.0	183.6 ± 17.4	231.3 ± 16.2	Invertivore	0-60	-	50	-	4, 7
Neolamprologus brevis	1	2	0	-	-	-	Invertivore	10-30	-	12	14	7
Neolamprologus brichardi	6	1	0	-	-	-	Invertivore	5-15	1.50	30	-	7, 20
Neolamprologus pulcher	3	11	12	58.1 ± 3.5	84.3 ± 5.9	35.8 ± 1.4	Zooplanktivore	3-45	-	28	10	6, 7, 18
Neolamprologus sexfasciatus	6	6	10	169.5 ± 26.5	56.9 ± 7.4	88.8 ± 3.7	Invertivore	0-15	-	1000	-	7, 8
Neolamprologus tetracanthus	6	9	14	147.1 ± 13.8	55.6 ± 3.7	79.3 ± 4.9	Invertivore	0-7	-	200	-	7, 9, 12, 13
Neolamprologus tretocephalus	3	4	0	-	-	-	Invertivore	0-7	1.60	459	130	5, 7, 8, 25
Opthalmotilapia boops	2	1	0	-	-	-	Algivore	0-7	-	30	21	4, 7
Opthalmotilapia nasuta	9	12	14	215.8 ± 11.1	334.9 ± 26.6	121.0 ± 3.1	Algivore	5-15	5.00	24	-	2, 7, 9
Opthalmotilapia ventralis	2	15	13	150.8 ± 5.3	182.9 ± 13.2	113.4 ± 2.4	Algivore	2-10	4.80	14	-	7, 9
Perissodus eccentricus	1	0	1	136.0	85.0	79.3	Scale eater	40-130	-	200	-	7
Perissodus microlepis	4	7	11	112.9 ± 8.6	71.4 ± 9.5	83.7 ± 3.2	Scale eater	1-15	1.70	109	51	7, 22
Petrochromis famula	5	8	12	436.5 ± 38.5	545.5 ± 31.7	168.2 ± 7.5	Algivore	0-7	6.40	17	30	7, 8, 9
Petrochromis orthognathus	11	7	14	165.1 ± 19.6	263 ± 35.4	114.4 ± 7.5	Algivore	1-20	6.00	27	30	7, 8, 9
Plecodus paradoxus	0	1	1	193.0	180.0	107.8	Scale eater	0-130	-	200	51	7, 8
Plecodus straeleni	0	3	3	107.3 ± 6.5	70.7 ± 6.2	95.1 ± 3.2	Scale eater	1-19	-	100	-	7
Pseudosimochromis curvifrons	0	12	12	326.1 ± 22.5	375.9 ± 46.1	172.9 ± 8.0	Algivore	0-7	4.50	7	-	7, 9
Reganochromis calliurus	0	1	1	187.0	72.0	84.3	Invertivore	15-100	2.20	104	12	4, 7, 8
Simochromis babaulti	3	2	0	-	-	-	Algivore	5-10	-	25	-	7
Simochromis diagramma	5	8	13	294.4 ± 20.8	288.1 ± 27.0	183.9 ± 6.9	Algivore	5-10	5.20	25	-	7, 9
Simochromis pleurospilus	6	10	16	118.5 ± 6.5	206.9 ± 20.3	97.4 ± 4.3	Algivore	0-15	-	25	28	7, 17
Spathodus erythrodon	1	3	0	-	-	-	Algivore	2-3	-	12	23	7
Spathodus marlieri	4	2	0	-	-	-	Algivore	1-5	3.50	15	-	7, 10
Tanganicodus irsacae	2	2	0	-	-	-	Algivore	2-3	3.50	11	25	7, 10
Telmaotchromis temporalis	5	9	14	64.1 ± 5.5	89.6 ± 7.0	45.1 ± 2.0	Invertivore	3-15	-	50	-	7
Trematocara macrostoma	0	6	3	84.0 ± 5.1	44.0 ± 1.0	36.1 ± 4.0	Invertivore	50-120	-	60	-	7
Trematocara unimaculatum	6	4	9	210.6 ± 31.1	75.9 ± 8.8	63.2 ± 5.4	Invertivore	0-120	1.00	60	-	3, 7
Triglachromis otostigma	4	3	1	61.0	64.0	41.5	Invertivore	10-50	1.00	100	12	3, 7
Tropheus brichardi	3	1	0	-	-	-	Algivore	10-15	4.60	12	-	2, 7
Tropheus moorii	11	8	13	214.6 ± 14.8	227.3 ± 18.0	121.0 ± 4.7	Algivore	2-3	6.30	12	33	7, 24
Tylochromis polylepis	0	14	12	128.8 ± 10.8	90.7 ± 3.5	119.1 ± 6.2	Invertivore	0-25	-	100	-	7
Variablichromis moorii	6	3	8	81.1 ± 7.3	98.4 ± 8.9	76.2 ± 3.2	Invertivore	1-10	-	100	98	7, 8, 15
Xenotilapia boulengeri	1	0	1	152.0	38.0	123.7	Invertivore	5-30	-	100	31	7
Xenotilapia flavipinnis	7	7	9	69.2 ± 3.7	32.9 ± 3.1	57.3 ± 2.3	Invertivore	1-30	2.95	40	28	7, 21
Xenotilapia melanogenys	3	16	13	139.5 ± 7.1	85.3 ± 5.3	90.8 ± 3.1	Invertivore	5-50	-	30	-	7
Xenotilapia ochrogenys	5	0	0	-	-	-	Invertivore	0-20	3.10	38	21	7, 9
Xenotilapia spiloptera	7	0	1	42.0	35.0	52.5	Invertivore	15-40	3.00	20	-	7, 8

Hypothesis tested	Sample size indiv./species	Parameter		Posterior mean	LCI	UCI	*p*
i) Direct metabolic constraints	707/71	Depth		0.005	-0.014	0.026	0.59
		Body mass		0.175	0.169	0.181	**< 0.001**
		Sex		0.008	0.001	0.015	**0.02**
ii) Expensive tissue	490/54	Gut		-0.013	-0.022	-0.003	**0.009**
		Depth		-0.006	-0.038	0.026	0.70
		Body mass		0.168	0.158	0.179	**< 0.001**
		Sex		0.011	0.003	0.020	**0.01**
		Guild	Inve.	0.046	-0.030	0.119	0.23
			Pisc.	0.064	-0.048	0.180	0.29
			Scal.	-0.033	-0.207	0.170	0.72
			Zoop.	-0.019	-0.151	0.108	0.75
iii) Expensive brain	461/43	Egg size		0.030	0.001	0.057	**0.04**
		Body mass		0.173	0.167	0.180	**< 0.001**
		Sex		0.004	-0.003	0.012	0.34
	707/71	Clutch size		0.015	-0.006	0.036	0.16
		Body mass		0.175	0.168	0.180	**< 0.001**
		Sex		0.008	0.001	0.016	**0.02**
iv) Brain malnutrition risk	373/37	Care duration		0.026	0.006	0.043	**0.007**
		Body mass		0.175	0.167	0.183	**< 0.001**
		Sex		0.004	-0.004	0.013	0.31

Table S2: A complete Bayesian statistics for multivariate models with phylogeny as random factors for our test for the expensive tissue hypothesis. Response variable is $\log_{10}$ transformed brain mass in both models. Sample size, the posterior mean with 95% credibility intervals (LCI: lower credible interval, UCI: upper credible interval) and the *p*-value for each parameter are presented. Note that the posterior mean for continuous variables (i.e. body mass, depth, and gut) represents the partial regression coefficient, while the posterior mean represents the differential intercept coefficient for nominal variables (i.e. sex and guild) compared with female in sex and algivore in guild respectively. Abbreviation of each levels of trophic guild represents Inve. = Invertivore, Pisc. = Piscivore, Scal. = Scale eater, Zoop. = Zooplanktivore, respectively. Statistically significant values are shown in bold font.

Table S3: Bayesian statistics for multivariate models without phylogenetic correction. Response variable is $\log_{10}$ transformed brain mass in both models. Sample size, the posterior mean with 95% credibility intervals (LCI: lower credible interval, UCI: upper credible interval) and the *p*-value for each parameter are presented. Even when we ignore phylogenetic information, care duration are positively correlated with brain size in both separate analysis (a) and multiple regression with other two life history traits (b).

	Sample size indiv./species	Parameter	Posterior mean	LCI	UCI	*p*
a)	373/37	Care duration	0.037	0.028	0.046	**< 0.001**
		Body mass	0.201	0.193	0.211	**< 0.001**
		Sex	-0.007	-0.026	0.001	0.43
b)	269/24	Egg size	0.045	0.034	0.057	**< 0.001**
		Clutch size	0.011	0.162	0.180	0.07
		Care duration	0.028	0.020	0.036	**< 0.001**
		Body mass	0.175	0.168	0.180	**< 0.001**
		Sex	0.008	0.001	0.016	**0.02**

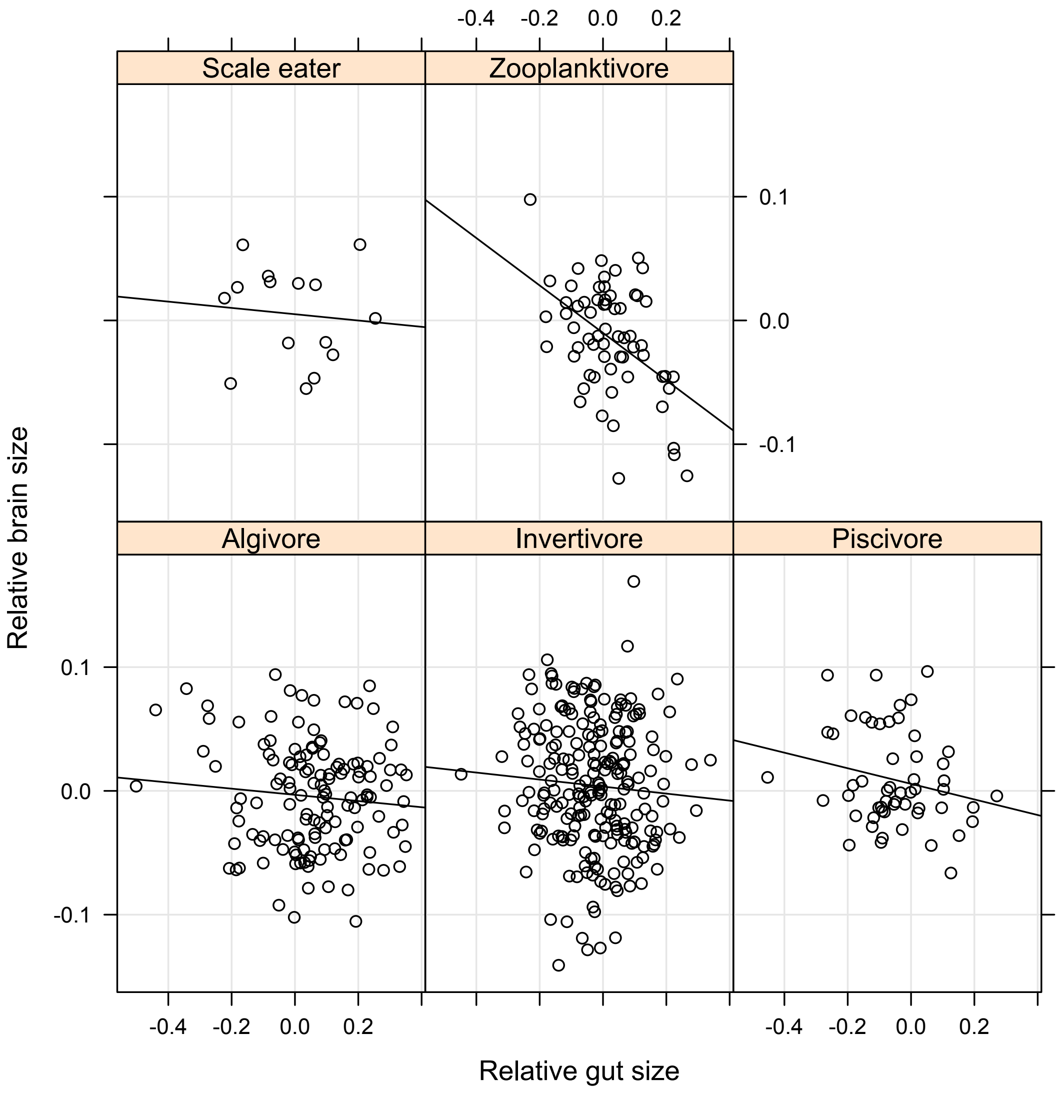

Figure S1: The relationship between brain and gut size, presenting each trophic guild separately. Values on the x-axis are residuals from a linear regression of $\log_{10}$ gut length as a dependent variable and $\log_{10}$ body mass, $\log_{10}$ depth, and sex as independent variables (relative gut size). Values on the y-axis are residuals from a linear regression of $\log_{10}$ brain mass as a dependent variable and $\log_{10}$ body mass, $\log_{10}$ depth, and sex as independent variables (relative brain size). The tribe to which each species are assigned to was included as a random effect for calculating values in both axes.

References for table S1

1. Awata, S. and M. Kohda, *Parental roles and the amount of care in a bi-parental substrate brooding cichlid: The effect of size differences within pairs.* Behaviour, 2004. **141**: p. 1135-1149.
2. Axelrod, H.R., *The most complete colored lexicon of cichlids: every known cichlid illustrated in color*. 1993: TFH.
3. Coulter, G.W., *Lake Tanganyika and its life*. 1991.
4. Froese, R. and D. Pauly. *FishBase*. 2011; Available from: http://www.fishbase.org.
5. Gashagaza, M.M., *Diversity of breeding habits in lamprologine cichlids in Lake Tanganyika.* Physiol. Ecol. Japan, 1991. **28**: p. 29-65.
6. Grantner, A. and M. Taborsky, *The metabolic rates associated with resting, and with the performance of agonistic, submissive and digging behaviours in the cichlid fish Neolamprologus pulcher (Pisces : Cichlidae).* Journal of Comparative Physiology B-Biochemical Systemic and Environmental Physiology, 1998. **168**(6): p. 427-433.
7. Konings, A., *Back to Nature Guide to Tanganyika cichlids*. 2nd Edition ed. 2005, El Paso: CichlidPress.
8. Konings, A., *Tanganyika cichlids in their natural habitat*. 1998, El Paso: Cichlid Press.
9. Kuwamura, T., *Parental Care and Mating Systems of Cichlid Fishes in Lake Tanganyika - a Preliminary Field Survey.* Journal of Ethology, 1986. **4**(2): p. 129-146.
10. Kuwamura, T., M. Nagoshi, and T. Sato, *Female-to-Male Shift of Mouthbrooding in a Cichlid Fish, Tanganicodus-Irsacae, with Notes on Breeding Habits of 2 Related Species in Lake Tanganyika.* Environmental Biology of Fishes, 1989. **24**(3): p. 187-198.
11. Morley, J.I. and S. Balshine, *Reproductive biology of Eretmodus cyanostictus, a cichlid fish from Lake Tanganyika.* Environmental Biology of Fishes, 2003. **66**(2): p. 169-179.
12. Nagoshi, M., *Survival of Broods under Parental Care and Parental-Roles of the Cichlid Fish, Lamprologus-Toae, in Lake Tanganyika.* Japanese Journal of Ichthyology, 1987. **34**(1): p. 71-75.
13. Nakano, S. and M. Nagoshi, *Brood Defense and Parental-Roles in a Biparental Cichlid Fish Lamprologus-Toae in Lake Tanganyika.* Japanese Journal of Ichthyology, 1990. **36**(4): p. 468-476.
14. Ochi, H., *Mating systems of two midwater-spawning cichlids, Cyprichromis microlepidotus and Paracyprichromis brieni, in Lake Tanganyika.* Ichthyological Research, 1996. **43**(3): p. 239-246.
15. Rossiter, A., *Lunar spawning synchroneity in a freshwater fish.* Naturwissenschaften, 1991. **78**(4): p. 182-184 %@ 0028-1042.
16. Sato, T., *Active accumulation of spawning substrate: a determinant of extreme polygyny in a shell-brooding cichlid fish.* Animal Behaviour, 1994. **48**(3): p. 669-678.
17. Taborsky, B. and K. Foerster, *Female mouthbrooders adjust incubation duration to perceived risk of predation.* Animal Behaviour, 2004. **68**: p. 1275-1281.
18. Taborsky, B., et al., *Stable reprogramming of brain transcription profiles by the early social environment in a cooperatively breeding fish.* Proceedings of the Royal Society B-Biological Sciences, 2013. **280**(1753).
19. Yamagishi, S. and M. Kohda, *Is the cichlid fish Julidochromis marlieri polyandrous?* Ichthyological Research, 1996. **43**(4): p. 469-471.

20. Yamaoka, K., *Trophic ecomorphology of Tanganyikan cichlids*, in *Fish communities in Lake Tanganyika*, H. Kawanabe, H. Michio, and M. Nagoshi, Editor. 1997, Kyoto University Press: Kyoto. p. 25-56.
21. Yanagisawa, Y., *Parental Care in a Monogamous Mouthbrooding Cichlid Xenotilapia-Flavipinnis in Lake Tanganyika.* Japanese Journal of Ichthyology, 1986. **33**(3): p. 249-261.
22. Yanagisawa, Y. and M. Nshombo, *Reproduction and parental care of the scale-eating cichlid fish Perissodus microlepis in Lake Tanganyika.* PHYSIOL. ECOL. JAP., 1983. **20**(1): p. 23-31.
23. Yanagisawa, Y. and H. Ochi, *Food-Intake by Mouthbrooding Females of Cyphotilapia-Frontosa (Cichlidae) to Feed Both Themselves and Their Young.* Environmental Biology of Fishes, 1991. **30**(3): p. 353-358.
24. Yanagisawa, Y. and T. Sato, *Active Browsing by Mouthbrooding Females of Tropheus-Duboisi and Tropheus-Moorii (Cichlidae) to Feed the Young and or Themselves.* Environmental Biology of Fishes, 1990. **27**(1): p. 43-50.
25. Yuma, M. and T. Kondo, *Interspecific relationships and habitat utilization among benthivorous cichlids*, in *Fish communities in Lake Tanganyika*, H. Kawanabe, H. Michio, and M. Nagoshi, Editor. 1997, Kyoto University Press: Kyoto. p. 87-103.
